# Every medicine is medicine; exploring inappropriate antibiotic use at the community level in rural Ghana

**DOI:** 10.1186/s12889-020-09204-4

**Published:** 2020-07-14

**Authors:** Samuel Afari-Asiedu, Marlies Hulscher, Martha Ali Abdulai, Ellen Boamah-Kaali, Kwaku Poku Asante, Heiman F. L. Wertheim

**Affiliations:** 1 Kintampo Health Research Centre, Ghana Health Service, Kintampo, Bono East Region Ghana; 2 Radboud University Medical Centre, Department of Medical Microbiology and Radboudumc Center for Infectious Diseases, Nijmegen, Netherlands; 3 Radboud University Medical Center/Scientific Center for Quality of Healthcare, Radboud University, Nijmegen, Netherlands; 4 Department of work and psychology, Maastricht University, Maastricht, Netherlands

**Keywords:** Antibiotics, Antibiotic use, Antibiotic resistance, Inappropriate antibiotic use, Ghana

## Abstract

**Background:**

Inappropriate antibiotic use is an important driver of antibiotic resistance. This study sought to explore inappropriate antibiotic use and confusing antibiotics with other medicines in Ghana using ethnomethodology research approach.

**Methods:**

This was an explorative study involving 15 in-depth interviews among health professionals and private dispensers and eight focus group discussions among 55 community members. Qualitative data were coded using Nvivo 12, thematically analysed and presented as narratives with quotes to support the findings.

**Results:**

Self-medication was common and antibiotics were used to treat specific diseases but respondents were not aware these were ‘antibiotics’. Various antibiotics were used for indications that in principle do not require systemic antibiotics, like stomach ache and sores on the body. Antibiotics, in particular tetracycline and metronidazole, were poured into “akpeteshie” (local gin) to treat hernia and perceived stomach sores (stomach ulcer). These practices were copied/learnt from various sources like over-the-counter medicine sellers, family, friends, radio/television, drug peddlers, pharmacies and doctors. Medicines in capsules were referred to as ‘topaye’ or ‘abombelt’ in Twi (local dialect) and perceived to treat pain associated with diseases. Antibiotics in capsules were described with colours which appeared confusing as some capsules with different drugs in them have similar colours.

**Conclusion:**

Inappropriate antibiotic use were influenced by general lack of knowledge on antibiotics and identification of antibiotics by colours of capsules which leads to confusion and could lead to inappropriate antibiotic use. There is the need for public health education on appropriate antibiotic use and standardization of appearance of antibiotics and other drugs to optimize use.

## Background

Globally, the upsurge in antibiotic resistance is largely attributed to inappropriate antibiotic use and poor infection control [1–3]. Though inappropriate antibiotic use is a global phenomenon [3], it is more prevalent in low and middle income countries (LMIC) especially in rural communities with poor access to approved healthcare providers [3, 4]. In LMIC, antibiotics are easily accessible and can be obtained without prescription from over-the-counter medicine sellers but also official pharmacies [5, 6]. Inappropriate antibiotic use is also associated with culture and community contextual factors such as selling or buying antibiotics without prescription and long distances to appropriate health care provider respectively [5, 7, 8].

Inappropriate antibiotic use includes various forms of use that are not in line with internationally accepted standards of care, including but not limited to: not completing the course, taking an insufficient dose, taking antibiotics for wrong indications and sharing antibiotics [9]. Antibiotics are medicines that should only be used to treat bacterial infections and not diseases caused by viruses such as influenza or common cold, but this is not adhered to [9].

In a recent study in central Ghana, we found that antibiotics were often used to treat non-bacterial infections. Also, there were indications that antibiotics were confused for other medicines with similar appearance, often capsules. In this study, we realized that there was a general confusion regarding how to recognize an antibiotic or how to call them which could contribute to inappropriate use [7, 8, 10]. Community members’ or patients’ ability to identify an antibiotic is therefore important for any future intervention aimed at improving antibiotic use. In Ghana, common antibiotics are in capsules, but also other medicines such as pain killers and traditional herbal medicine. Amoxicillin and Tetracycline are generally referred to as ‘red and yellow’ which may be confusing as both capsules have similar colours and could be used interchangeably [7, 8]. Local cultures such as buying and using antibiotics based on previous experience and using antibiotic based on friends or family members recommendations have been developed around the use of some antibiotics [8].

This study sought to further explore inappropriate antibiotic use and confusing antibiotics with other medicines which are in capsules, tablets and antibiotics with similar colours at the community level in Ghana. Such understanding will inform the design of interventions to educate the community on medication use including antibiotics.

## Methods

The methods for this study are reported according to the consolidated criteria for reporting qualitative research framework (COREQ) [11, 12].

### Theoretical perspective

The conduct of this study was guided and explained with an ethnomethodology research approach. Ethnomethodology is a phenomenological approach to interpret everyday action/behaviour in a social context [13, 14]. The approach aims to guide research into meaningful practices and everyday activity as experienced by participants. The key objective of the approach is to understand the rules that underlie everyday activity-in this case medicine use, including antibiotic use and thus constitute part of the norms of a given society. Research from this perspective generally focuses on ordinary and routine forms of behaviour. The approach emphasizes the contextuality of practice thus the richness of shared understandings that guide and orient participants’ actions in a given practice or activity [14, 15].

In this study, we explored the contextual factors that underlie inappropriate antibiotics use. We explicitly focused on local cultures that have developed around the use of specific antibiotics; thus the type of antibiotics that were mentioned by respondents and the diseases they treat. The application of this approach in this study is important because human behaviour is influenced by immense background knowledge as used by people in daily life; thus the meaning individuals ascribe to their behaviours is based upon a stock of previous experiences. For instance, community members who have previously experienced the effectiveness of some antibiotics tend to self-medicate with the same or similar antibiotics when they are sick or have similar symptom [8, 16].

### Study design

This was an explorative study conducted between January and March, 2019. Using ethnomethodology research approach, in-depth interviews (IDI) and focus group discussions (FGDs) were conducted with health professionals and dispensers in hospitals, private pharmacies and licenced chemical sellers (LCS)/over-the-counter medicine sellers (OTC) shops. IDIs were first conducted with health professionals and other dispensers to understand their perspective on inappropriate antibiotic use by community members. Findings from the IDI were used to refine the FGD guide to further explore inappropriate antibiotic use among community members. Issues about confusing antibiotics with other medicines in capsules and tablets were further explored during the FGDs.

### Study area

Data was collected in Kintampo North and South Districts which are located within the forest-savannah transitional ecological zone in the Bono East Region of Ghana. The study area covers an area of 7162 km^2^ with a resident population in 2016 approximately 156,145 [17]. The study setting is largely rural and subsistence farming is the major occupation. Most inhabitants initiate treatment of ailments at home, then continue to the LCS/OTC to buy medicines including antibiotics, and they may finally end up in a health facility if their illnesses do not resolve [18]. There are two public district hospitals, 12 health centers/clinics and 30 Community-based Health Planning and Services (CHPS) compounds. For private health facilities, there are 4 clinics, 2 maternity homes, 4 pharmacies, and 86 LCS/OTC. These facilities provide health services to urban and majority of rural poor community members.

### Data collection

Data was collected on antibiotic use for diseases including using antibiotics for wrong indications and/or for non-microbial infections, how people learn about antibiotics and indications for use and confusion of antibiotics with other medicines.

#### IDI with health workers and licensed chemical sellers/over-the-counter medicine sellers

Fifteen IDIs were conducted among health workers and dispensers of medicines. As indicated earlier, in our previous study we found that antibiotics were confused for other medicines with similar appearance, often capsules [7, 8]. Guided by findings from these studies, the IDI guide for health workers was co-designed by the research team (i.e. the first author, two colleague authors and 3 senior authors of this paper who are experts in this field) and piloted with three respondents to test for its appropriateness. During the pilot respondents were asked explicitly whether every question was clear, and whether they felt that everything they wanted to share was explored. Based on the pilot responses, the data collection team (i.e. the first author and two colleague authors of this paper) debriefed and adjusted the guide by adding further probing questions to enhance that different respondents understand each question in a similar way. The revised guide was reviewed by the research team and approved for data collection. This process of fine-tuning the IDI guide was done to ensure that the guide was valid and evoked reliable responses. The pilot IDIs provided good information which was added and analysed as part of the data collected. The IDIs sought to understand the perspectives of health workers on inappropriate antibiotic use by their patients/clients and on patients’ confusing of antibiotics with other medicines in capsules, tablets and antibiotics with similar colours. Respondents included two medical doctors, one pharmacists, two physician assistants, one nurse, one midwife, and two dispensing technicians in the two district hospitals (Table 1). IDIs were also conducted with two dispensers at private pharmacies and 4 LCS/OTC. Respondents were purposively selected to represent categories of health professionals and dispensers who prescribe and dispense antibiotics in the study area. Respondents were 18 years or older.

**Table 1:** Summary of IDI and FGDs

Respondents	No. of respondents
***IDIs for Health professionals and dispensers at private pharmacy and LCS***	
Medical Doctors	2
Pharmacists	1
Physician assistants	2
Nurses/Midwives	2
Dispensing technicians (district hospitals)	2
Dispensing technicians (private pharmacies)	2
Licenced chemical sellers/over-the-counter medicine sellers	4
***Community members FGDs***	
Women caretakers of children under five years (group 1)	6
Women caretakers of children under five years (group 2)	6
Women caretakers of children under five years (group 3)	6
Women caretakers of children under five years (group 4)	6
Men (group 1)	8
Men (group 2)	10
Men (group 3)	7
Men (group 4)	6
**Total**	**70**

#### Community member FGDs

Eight FGDs (four with women caretakers of children under 5 years and four with men who were generally household heads) were conducted among selected community members (Table 1). After the initial design of the FGD guide based on findings from our previous study, preliminary findings from IDI were used to refine the guide. Similar to the procedures for the IDI guide, an FGD guide was developed, piloted with one group and adapted before using it in the study population. Data from the pilot FGD was subsequently added and analysed together with the rest of the data collected. Issues about confusing antibiotics with other medicines in capsules and tablets were further explored during the FGDs. Community members, 18 years old and above were purposively selected to participate in this study. Women caretakers of children under 5 years attending child welfare clinic at the district hospitals were contacted. The men were also contacted by field staff who work in the study area in their communities. The purpose of the study were explained to participants who were contacted for the study and those who were willing to participate were included in the study. The main themes explored included medicine use including antibiotics, specific diseases for which antibiotics were used and reasons for using such antibiotics as well as confusing antibiotics with other medicines in capsules and tablets.

The numbers of IDIs and FGDs conducted were guided by the principle of saturation. For the IDIs we realized responses from the eleventh interview through to the fifteenth were similar indicating saturation has occurred. During the FGDs, we noticed the point of saturation by the seventh session but we continued with one other group we had already prepared. The occurrence of saturation during the IDIs and FGDs where subsequent responses were similar to responses in earlier interviews further confirmed the reliability of the study guides.

#### Study procedures for IDIs and FGDs

Eligible participants were informed about the purpose, procedures, and anonymity of the interview. IDIs were conducted face-to-face in Twi (widely spoken local dialect in the study area) or English in the premises where medicines were dispensed to patients/clients. IDIs were conducted between 3:00 pm to 5:00 pm GMT when attendance was very low to avoid interruption. IDIs lasted for about 45 min. FGDs were conducted in Twi between 8:00 am to 10 am GMT in a place of convenience for respondents and lasted for about 60 min. Each FGD session was made up of 6 to 8 participant who sat in semi-circular manner. Participants were numbered serially from one to between six and eight according to their sitting positions and first called their numbers when responding to questions. IDIs and FGDs were audio recorded and conducted by a moderator (first author); an experienced qualitative researcher with a master’s degree in Sociology and a note-taker with a Bachelors in Public Health. The moderator stood in front of the group and placed the audio recorder on a table in the middle of the group. The note-taker sat beside the moderator to observe proceedings whilst taking notes from the discussion. The moderator used common antibiotics and other medicines in capsules and tablets for demonstration during discussions. IDIs and FGDs were conducted with an interview guide made up of predetermined questions and themes. The interview guide specified the exact questions the moderator asked under the various themes, along with follow-up questions. However, as part of probing, other related emerging issues that were initially not included in the interview guide were also discussed as and when they emerged. Interview sessions were brought to an end when the moderator had exhausted all questions on the interview guide and on other emerging issues.

### Data management and analysis

Data management and analysis was guided by thematic analysis approach [19]. Audio recordings of IDIs and FGDs were transcribed verbatim into English language. The transcripts were thoroughly read and edited where necessary to ensure that they reflect the audio recordings. The transcripts were uploaded into a qualitative data management and analysis software (NVivo 12) for coding. In Nvivo, a priori themes (ie antibiotic use for specific diseases, how people learn antibiotics will cure specific diseases, and confusion about antibiotics) were created to guide the coding of transcripts. Whiles coding the data, sub-themes were created to inductively capture emerging issues. After coding the responses into nodes (themes and sub-themes), the coded response were thoroughly read and where appropriate, responses were uncoded and recoded. In some instances, responses were discussed among team members before uncoding and recoding. This was followed by an interpretive analysis of collated responses under various theme. The findings were presented as narratives and supported with excerpts or quotes from the respondents.

## Results

This section is categorized into four sections: (i) demographic characteristics of the respondents (ii) antibiotic use for specific diseases (iii) how people learn about antibiotics and indications for use and (iv) confusion of antibiotics with other drugs.

(i) **Demographic characteristics of IDI and FGD respondents.**

A total of 15 health professionals/dispensers including medical doctors, pharmacist, midwives/nurses, physician assistants, dispensing technicians and LCS/OTC participated in the IDI sub-study. Eleven were males and 4 were females between the ages of 32 to 50 year. Fifty-five community members participated in the FGD sub-study, of which 31 (64%) were males and 24 (44%) were females. Community members who participated in the FGD were between the ages of 20 to 50 years. Thirty community members (55%) were traders/farmers.

(ii) **Antibiotic use for specific diseases.**

Antibiotics were used to treat diseases including stomach pain, diarrhoea, gonorrhoea, wounds/sores, boils and headache. Many of these indications are not caused by bacteria, which was also confirmed by health professionals as common in the study area.

*They abuse antibiotics; the most abused one is Amoxicillin. Because of headache someone will go to the drug store [LCS/OTC] and say s/he wants to buy Amoxicillin. (IDI with Medical Officer #2).*

*We know that antibiotics like Chloramphenicol and Tetracycline are used by some people to treat sores. Sometimes, when patients/clients have abdominal pain, they buy Tetracycline* (IDI with Physician Assistant *#*1).

### Antibiotics for stomach pain, diarrhoea, gonorrhoea, cold

Amoxicillin, Tetracycline, Metronidazole and Chloramphenicol were generally used to treat stomach aches which are not considered to be caused by bacterial, unless it concerns gastric ulcer (H pylori infection). Amoxicillin and Tetracycline are also used for gonorrhoea which is a bacterial infection (sexually transmitted infection).

*I had stomach pain and the LCS/OTC attendant told me to buy the red and yellow [tetracycline] capsules and flagyl [metronidazole] because I have stomach ulcer. So anytime I realize that I am about to feel stomach pain, I take the red and yellow [Tetracycline.* (FGD#2 with males, respondent *#*10).

*Amoxicillin and Tetracycline are used by people having gonorrhoea. When they go to the drug store (LCS/OTC) they can buy the ‘red and yellow’ [Tetracycline] and Amoxicillin, and take two capsules each.* (FGD#4 with males, respondent *#*1).

*The common one is amoxicillin and some of them use it for common cold and sometimes stomach pains.* (IDI with a dispensing technician at a district hospital_ respondent *#*1).

Stomach pain is generally attributed to having sore in the stomach [stomach ulcer]. It is therefore believed that once Amoxicillin, Tetracycline and Metronidazole could cure sores on the surface of the body, it could equally cure the sore in the stomach and vice-versa.

*It’s our own thinking; you know that when you are experiencing pains in your stomach, it is sore that has developed in your stomach so you can take the tetracycline or the white antibiotic [chloramphenicol]. This is because it’s the same way that, when the sore develops on your body, you will use same tetracycline or white antibiotic [chloramphenicol].* (FGD#4 with males, respondent *#*1).

These antibiotic are not taken according to any dosing regimen or instruction considering that most of them are purchased without prescription from the pharmacy, LCS shops and drug peddlers;

*For medicine we get from the drug stores [LCS/OTC], as soon as you start taking them and you feel you are okay, you leave the rest.* (FGD#1 with males, respondent *#*2).

*I take 2 tablets of the Flagyl [Metronidazole], and 2 of the Amoxicillin. The Flagyl is really bitter so what I do is to mash it with stone before taking since I cannot chew it due to its bitterness.* (FGD#3 with males, respondent *#*1).

### Antibiotics for treating wounds or sores

Chloramphenicol, Amoxicillin, Tetracycline, Metronidazole and Penicillin V are used for the treatment of sores on the body;

*These two capsules [pointing at tetracycline and Chloramphenicol]; if someone has a sore and the person pour this one [tetracycline] into the sore and it doesn’t get healed, the person will change and use the white one [Chloramphenicol].* (FGD#4 with males, respondent *#*1).

These antibiotics are generally poured into the sore in different ways until the sore gets healed or antibiotics are changed if it does not work as expected.

*When I recently got hurt, I poured four of the [Chloramphenicol] and mixed it with two of [Tetracycline] and I change it every 3 days for about a month.* (FGD#3 with males, respondent *#*1).

*You can mix three capsules of Chloramphenicol with shea butter and rub it on the wound.* (FGD#8 with mothers of under-five children *#*8).

*Antibiotics can be poured on sores to heal them; even flagyl [metronidazole] we grind it and pour it on the sore to heal it.* (FGD#2 with males, respondent *#*10).

### Antibiotics for boils

Cloxacillin and Flucloxacillin were generally mentioned as medicines that are used for the treatment of boils.

*Cloxacillin and Flucloxacillin are basically for boils.* (FGD#3 with males, respondent *#*1).

*Sometimes when you have boils, they can give you the Flucloxacillin to reduce the swelling and pain.* (FGD#6 with mothers of under-five children, respondent #6).

### Antibiotics used in alcohol to treat hernia and sores in the stomach

While some respondents mentioned that they have never heard or seen such practice, others responded affirmatively and mentioned categorically the types of antibiotics which are poured into “akpeteshie” [a local gin distilled from palm tree] to treat hernia and sores in the stomach;

*It’s true that some people pour antibiotics in akpeteshie and drink it to treat hernia.* (FGD#4 with males, respondent *#*5).

*Some people also pour tetracycline into “akpeteshie” and take. People prefer to use the [tetracycline] than the Flagyl [metronidazole] because the Flagyl [metronidazole], is bitter.* (FGD#3 with males, respondent *#*5).

It also emerged that pouring antibiotics into akpeteshie is not only practiced by people who drink alcohol but some women do it after delivery to heal the sore in their wombs.

*It’s not only drunkards who pour tetracycline into alcohol to drink to treat hernia; when some women deliver, they pour Chloramphenicol into the alcohol to heal the sore in their womb.* (FGD*#*3 with males, respondent*#*4).

(iii) **How people learn antibiotics will cure specific diseases.**

There were five ways by which respondents learn about medicines including antibiotics and the diseases they are perceived to treat. First, respondents learn about medicines including antibiotics prescribed in hospitals and other health facilities or dispensed by pharmacies and LCS/OCT for their diseases and subsequently engage in self-medication with the same antibiotics when they experience similar symptoms. Second, respondent self-medicate with medicines including antibiotics based on recommendations of family relatives, colleagues and friends. Third, others learn from radio and television adverts. Four, drug peddlers who move from house-to-house with medicines also teach people the use of specific antibiotics. Five, some respondents learn about antibiotic use based on assumptions. As specified earlier, Amoxicillin, Tetracycline and Metronidazole were used to treat stomach ulcer based on the assumption that once these antibiotics could cure sores on the body, it could equally cure sore in the stomach. The responses below corroborate these findings;

*Let’s say I am having stomach pains and I go to the hospital and the doctor prescribes Flagyl [metronidazole] for me. The next time I have the same pain [symptom] I will just go to the drugstore (LCS/OTC) to get the Flagyl.* (FGD#4 with males, respondent *#*1).

*This man [pointing at a participants] is my friend and my friend also has a friend. If he was sick and somebody told him [Chloramphenicol and tetracycline] are used to treat stomach ache and my friend hears that, the next time another person also has stomach ache he will also tell that person to go and buy the [Chloramphenicol tetracycline]...that is how all of us get to know about the medicines.* (FGD#2 with males, respondent *#*5).

*When I was little, I remember some people use to move from house to house with drugs, they taught me what the drug does. Especially with the [Amoxicillin] and [Chloramphenicol].* (FGD#5 with mothers of under-five children *#*2).

(iv) **Confusion about antibiotics.**

Generally, community members do not know what antibiotics are and the diseases they should be used for. Though antibiotics are used to treat bacterial diseases, respondents do not know that they are antibiotic per se and that they should be used to treat specific infectious diseases. Medicines that are in capsules referred to as ‘Topaye’ or ‘Abombelt’ in Twi (the local dialect widely spoken in study area) including antibiotics are perceived to treat specific types of diseases.

*“Topaye” is any medicine that is powder and put into tiny rubbers [capsules] and can’t be taken in its powder form but when it gets into your stomach and water pours on it, it will burst, that is why it is called Tupaye [throw and burst].* (FGD#3 with males, respondent *#*3).

This was corroborated by health workers:

*They [patients] call all of them capsules; capsules in the sense that it is covered and when you take it inside [stomach], it burst. That’s why it is called capsules [literally, throw and burst].* (IDI with a midwife).

### Confusing antibiotics and pain killers

Medicines which are in a capsule (antibiotic or painkiller) are taken to treat pain associated with certain diseases. It emerged that the primary reason for taking antibiotics is to treat the pain associated with diseases but not specifically the infectious cause, even though the pain could be a result of an infectious disease.

*Patients think “capsules” is medicine which relieves pain associated with diseases. That’s why when they get abdominal pain, they take it. So they take “capsules” as pain medication. Antibiotics in capsules is the most consumed antibiotics by the public. They take the “capsule” to reduce the pain but they don’t know its antibiotic.* (IDI with a nurse).

Most of the common antibiotics which are easily accessible in the study area, some painkillers and other medicines are also in capsules as such they are considered as medicines in the same category.

*Painkillers such as diclofenac, tramadol and even brufen are usually in capsules depending on the company likewise the common antibiotics.* (IDI with Physician Assistant *#*1)

*Most of the common antibiotics are in capsules except the stronger generic like Amoxicillin/clavulanic acid which is in tablet. Amoxicillin, Doxycycline etc are in capsules. We have this one, tramadol capsules which is a painkiller but it is in capsules.* (IDI with Medical Officer *#*1).

### Confusing antibiotics due to colours

Locally, medicines including antibiotics were identified or described with colours because people do not know the names of these medicines. Antibiotics were identified and referred to with colours such as red, red and yellow, white, green, black capsules.

*So we have the red capsule [Tetracycline], white capsule [Chloramphenicol] and we consider [flucloxacillin, cloxacillin] as black capsules.* (FGD#3 with males, respondent *#*3).

The identification of antibiotics with colours appeared to be confusing considering that some antibiotics in capsules have similar colour. Typical examples are Amoxicillin and Tetracycline which are both in red and yellow capsules. There was some level of consensus among community members and dispensers at LCS/pharmacy that tetracycline is referred to as “red and yellow” and Amoxicillin is called by its name.

*We are used to mentioning the amoxicillin as compared to the other one [Tetracycline]; we just call it red and yellow because we are not used to the name.* (FGD#4 with males, respondent *#*5).

*Its tetracycline they are referring to when they say red and yellow, the light red and yellow is what they called amoxicillin. They are never confused about colours.* (IDI with a dispensing technician at a private pharmacy_ respondent *#*1).

Some community members mentioned that the colours of Amoxicillin and Tetracycline are confusing.

*Actually the colours of these two drugs [Amoxicillin and tetracycline] are confusing. [Tetracycline] has been in the system for a very long time since my childhood before [Amoxicillin] was introduced.* (FGD*#*1 with males, respondent *#*3).

Also, there was evidence of disagreement as to which medicines (tetracycline or amoxicillin) is really referred as ‘red and yellow’ among health professionals.

*When patients say red and yellow they [patients] mostly refer to amoxicillin but amoxicillin is not the only red and yellow coloured drug and that’s the problem.* (IDI with Medical Officer *#*1)

*Yes! It is the tetracycline they [patients] refer to as red and yellow, but for some people when you put the tetracycline and amoxicillin side by side the person gets a bit confused.* (IDI with a midwife)

### The use of amoxicillin and tetracycline interchangeably

Whilst some community members indicated that Amoxicillin and Tetracycline were the same others emphasized that the two antibiotics were used to treat different disease conditions.

*The two [Amoxicillin and Tetracycline] are the same. I have ever sent a child to go and buy “red and yellow” [Tetracycline] on about three occasions and the child returned with Amoxicillin. When I followed up I was told they treat the same illness.* (FGD#1 with males, respondent *#*10).

*Amoxicillin and Tetracycline are for different purposes; each medicine is for a specific purpose even though they are both antibiotics.* (FGD#6 with mothers of under-five children *#*3).

Some health professionals and community members were of the view that amoxicillin and tetracycline were sometimes used interchangeably due to the similarity in colours.

*It is true that people do mix them up especially the older people. When they send you to buy [Amoxicillin] and buy [Tetracycline], they can hardly see the difference.* (FGD#1 with males, respondent *#*8).

*They even mix them [Tetracycline and Amoxicillin] for instance; they [patients] take two capsules of each instead of two capsules of only one. They use them interchangeably because they don’t know the names, the description given can result in purchasing the wrong one.* (IDI with Licenced chemical seller *#*1).

Contrary to the above, some community members mentioned that though the colours of Amoxicillin and Tetracycline are both “red and yellow”, the colours are distinct and cannot be used interchangeably.

*Amoxicillin and Tetracycline cannot be used interchangeably because the red and yellow of Tetracycline is deep and that of Amoxicillin is light. For the Tetracycline people really know it so if you mistakenly give them Amoxycillin they will know*. (FGD#2 with males, respondent *#*3).

## Discussion

This study sought to explore inappropriate use of antibiotics and to provide insight into confusing antibiotics with other medicines at the community level in Ghana. Findings from this study are in line with the principles of ethnomethodology research approach considering that this study focused on ordinary and routine forms of behaviour with regards to the meaning which underlies inappropriate antibiotic use. Specific findings on diseases that antibiotics were perceived to cure and description of antibiotics with colours illustrates the context of practice and shared understandings which influence antibiotic use in the study area. This study further revealed local cultures that have developed around the use of specific antibiotics.

Self-medication emerged as a major contributing factor to inappropriate antibiotic use. Within the context of self-medication, antibiotics were used to self-treat stomach pain, diarrhoea, gonorrhoea, wounds, boils and hernia some of which may not have been caused by bacterial infection. Similar to findings from other studies, the challenge of self-medication thrives in our study context because there are no clear routes to healthcare seeking. This is due to the existence of multiple healthcare providers including LCS/OTC medicine sellers and drug peddlers whose medicine dispensing practices are unapproved [5, 8, 16] and has been documented as one of the major driver of inappropriate antibiotic use and resistance in LMICs [6, 16]. Consequently, without public health educational intervention, the problem of self-medication will continue to exist as community members learn about these practices/habits through structures within their context.

There was a general lack of knowledge on what antibiotics are and for what specific diseases they should be used among community members who are not health professionals. Antibiotics were considered just as any other medicine which relieves pain associated with diseases. Therefore the main reason for taking antibiotics in the context of this study was to relieve pain but not to treat bacterial infections. This findings is comparable to findings from a study in Pakistan where respondents were not conversant about the difference between “antibiotics” and the word “medicine” [20]. Similarly, in our study community members did not know the difference between antibiotics and other medicines. Antibiotics and other medicines are both in capsules or tablets and therefore considered as “medicine” in the same category considering that both relieves pain associated with diseases. Ethnomethodologically [13–15], the lack of knowledge about the differences between antibiotics and other medicines contributes to the inappropriate practices and use of medicines in general and specifically inappropriate antibiotic use.

Our findings further indicate that antibiotics were referred to by the colour codes of the capsules which may cause confusion confusing. This is because first, as indicated earlier, community members do not know what antibiotics are and second, the name ‘antibiotics’ is not in any of the local languages or there are no words or concepts in local languages in Ghana which specifically define ‘medicines that are used to treat infectious diseases caused by bacteria’. Specific antibiotics were therefore differentiated from other antibiotics with colours and the specific diseases they were perceived to treat. A name or concept of antibiotics in a widely spoken local language could help community members to understand and appreciate what antibiotics are and the diseases they treat; this will reduce identification of antibiotics with colours which contributes to inappropriate use. In line with the core principle of Ethnomethodology [13–15], the use of colours to describe antibiotics has become part of the rules which underlie everyday use of antibiotics. In other words, the use of colours to describe antibiotics is a contextual (ethno) method (methodology) which is used to negotiate antibiotics use and thus constitute part of the norms that have been developed around antibiotic use over a period of time. This finding could also be explained within the context of symbolic interactionism which is the process of interaction in the formation of meanings for individuals [21–23]. The use of specific colours to describe antibiotics is a socially agreed upon way of identifying these medicines and this has become the reality through the process of interaction. The colours used to identify antibiotics are socially agreed upon because community members largely share in the meaning of the colours and the types of antibiotics associated with them. This finding further emphasize the need for the development of a common local language to accommodate important words such as ‘antibiotics’ because language is an important tool which helps individuals to understand the meaning ascribed to things [21, 23].

Also, we found that medicines in capsules which are referred to as “topaye” in Ghana does not always mean antibiotics. The word “topaye” refers to medicines in capsules which could either be antibiotic or any other medicine. This to a large extent could contribute to inappropriate antibiotic use as people act towards objects based on the meanings they ascribe to those objects as espoused in symbolic interactionism [21]. As explained above, antibiotics are taken just as any other medicines to treat pain but not infections because of the meaning ascribed to them. Similarly there was the assumption that tetracycline, amoxicillin and chloramphenicol could treat wounds in the stomach because they are able to treat wounds on the body. This assumption is based on the interpretation of meaning ascribed to wound healing within or on the body. This finding shows that sometimes people use medicines based on their own ideas about safety and efficacy. For instance, red medicines are thought to be good for the blood in Sierra Leone [16, 24]. It is therefore important to note that this finding brings some clarity to bear on the widely held notion that “topaye”, which means capsules, is antibiotic. This view is probably due to the fact that most common antibiotic which are easily accessible at the LCS/OTC are usually in capsules as such “topaye” may have come to be synonymous with antibiotics.

There was some level of agreement among community members that tetracycline is generally referred to as red and yellow whilst amoxicillin is called by its name. On the other hand, health professionals confuse the two (Tetracycline and Amoxicillin) thus they are not sure which antibiotic patients really refer to as red and yellow. It is interesting to note that dispensers at LCS/OTC, pharmacies and drug peddlers shared the same meaning and understanding with the community members. This is so because LCS/OTC, pharmacies and drug peddlers are easily accessible and are more connected to community members through day to day interaction which is emphasized in ethnomethodology [13–15]. Health professionals should therefore be more involved in community practices especially medicine use for a holistic healthcare provision.

Our findings also indicate that community members learn about antibiotic practices and the choices of antibiotics used for specific diseases through structures within their context. This takes place at different levels of interaction i.e. through the health system (formal and informal) and individuals, friends and family. These practices acquired through socializations are passed on from generation to generation and consequently becomes the rules which underlie practices around the use of specific antibiotics. A typical example is the use of antibiotics in alcohol; though the source of this practice is not clear, it appears it came about through individual experimentation which has been learnt by others through interactions. Similar to other practices around antibiotic use, the use of antibiotics in alcohol is also a social behaviour which was learnt through the process of interaction among people [22].

### Limitation of study

During the FGDs antibiotics were explained in Twi as medicines which are used to treat bacterial infections. Twi is the widely spoken local language in the study area but it is not the first language for all inhabitants. Also, considering that, there is no specific word in Twi for antibiotic it was quite difficult explaining what antibiotics are. Also, it was therefore quite tedious explaining the difference between bacteria, virus and parasite taking cognisance of the fact that some antibiotics also have action on parasite. We therefore anticipate that not all respondents may have fully understood the terminologies we used in explaining what antibiotics are. However, this was mitigated by the use of common antibiotics and other medicines in capsules and tablets for demonstration during discussions. It is also important to state that this study explored contextual issues around inappropriate antibiotic use among purposefully selected respondents in two districts of Ghana. The findings of this study are, therefore, not generalizable though many insights might be transferable to similar settings.

## Conclusion

The general lack of knowledge on antibiotics and the perception that antibiotics are just like any other medicine which are taken to treat pain associated with diseases appears to be a contributing factor to inappropriate antibiotic use. Inappropriate antibiotics use could also be influenced by identification of antibiotics by colours of the capsules and the specific diseases they are perceived to cure. Some of the colours of antibiotics were similar especially tetracycline and amoxicillin and this created confusion in the identification which could lead to the interchangeable use of these antibiotics. Findings from this study therefore provide context and inform the design of tailored public health education on the appropriate antibiotic use. Public health educational messages should be crafted taking special cognisance of local dialects considering that language is an important tool for negotiating the meaning we ascribe to objects; in this context medicines and antibiotics. Our findings further indicate the need to consider standardizing the colour codes of common antibiotics to improve use. Common colour codes or other ways to harmonize the appearance of antibiotics and a clear labelling of antibiotics may empower community members to be conscious about antibiotics use and enable them to put future public health education about antibiotics into good practice.

## Data Availability

Data are available from the authors upon reasonable request.

## Abbreviations

FGD: Focus Group Discussions
IDI: In-depth Interviews
LMIC: Low and Middle Income Countries
LCS: Licenced Chemical Sellers
OTC: Over-the-counter medicine sellers
